# Molecular basis of acyl-CoA ester recognition by α-methylacyl-CoA racemase from *Mycobacterium tuberculosis*

**DOI:** 10.1016/j.jbc.2025.110302

**Published:** 2025-05-29

**Authors:** Otsile O. Mojanaga, Timothy J. Woodman, Matthew D. Lloyd, K. Ravi Acharya

**Affiliations:** Department of Life Sciences, University of Bath, Bath, United Kingdom

**Keywords:** α-Methylacyl-CoA racemase (AMACR, P504S), CoA-transferase, fenoprofen, ibuprofen, *M. tuberculosis*, X-ray crystallography, kinetic study

## Abstract

α-Methylacyl-CoA racemase (AMACR; P504S) enzyme plays a vital role in branched-chain fatty acid metabolism by catalyzing the conversion of 2-methyl-branched fatty acyl-CoAs into a near 1 to 1 mixture of the (2*R*)- and (2*S*)-epimers, enabling further metabolism. α-Methylacyl-CoA racemase from *Mycobacterium tuberculosis* (MCR) has been explored as a model to understand the AMACR racemization mechanism and as a drug target. Here we present a detailed analysis of a new MCR wild-type crystal structure to provide insights into the MCR racemization mechanism and the molecular features that contribute enzyme activity and selectivity. Specifically, we report a structure of wild-type MCR (in tetragonal space group I422, a new crystal form) along with 12 structures of MCR in complex with branched-chain 2-methylacyl-CoA esters (ibuprofenoyl-CoA, ±-fenoprofenoyl-CoA, *S*-ketoprofenoyl-CoA, ±-flurbiprofenoyl-CoA, *S*-naproxenoyl-CoA, *S*-2-methyldecanoyl-CoA, and isobutanoyl-CoA) and straight-chain acyl-CoA esters (decanoyl-CoA, octanoyl-CoA, hexanoyl-CoA, butanoyl-CoA, acetyl-CoA) in the range of 1.88 to 2.40 Å resolution. These detailed molecular structures enhance our understanding of substrate recognition and, coupled with extensive enzyme inhibition assays, provide a framework for the rational structure-based drug design of selective and potent MCR inhibitors to combat *M. tuberculosis* in the future.

Branched-chain fatty acids are common in the diet and are produced endogenously from cholesterol (1, 2). Degradation of branched-chain fatty acids occurs as the corresponding 2-methylacyl-CoA esters by β-oxidation. The β-oxidation pathway is stereoselective for *S*-2-methylacyl-CoA esters, but *R*-2-methylacyl-CoA esters are derived from dietary fatty acids or bile acids produced by the oxidation of cholesterol. Degradation of *R*-2-methylacyl-CoA esters is enabled by α-methylacyl-CoA racemase (AMACR; P504S), which catalyzes the conversion of *R*-2-methylacyl-CoAs to their corresponding *S*- epimers. The enzyme is also important in the pharmacological activation of ibuprofen and related drugs (1, 2).

In *Mycobacterium tuberculosis* (which causes tuberculosis), cholesterol metabolism contributes to its pathogenesis (3). Cholesterol derivatives can be converted to their corresponding bile acids by the action of cytochrome P_450_ enzymes, which are converted to their corresponding acyl-CoA esters. The 25*R*-methylacyl-CoA requires conversion to the 25*S*-methylacyl-CoA epimer before β-oxidation can occur (3) [the α-carbon bearing the methyl group is carbon-25 in the standard numbering system for steroids (2)]. *M. tuberculosis* encodes for three α-methylacyl-CoA racemases (*mcr*, *far*, *Rv3727*) (4), and MCR has been implicated in the epimerization of bile acid acyl-CoAs derived from cholesterol (3). MCR is catalytically active using isotope-exchange assays (5, 6, 7) and colorimetric assays (7). The structure of FAR has also been determined and shows that the enzyme is also a member of the family III CoA transferases (8), but enzymatic activity has not been confirmed.

Protein levels (1, 2) and activity (9, 10) of human AMACR (P504S) are increased in prostate cancer and other cancers, and the enzyme is a recognized drug target. Knockdown of human AMACR in prostate cancer cell lines reduces proliferation (9, 11, 12) and is synergistic with androgen deprivation (9). A number of acyl-CoA inhibitors of human AMACR 1A have been reported (Fig. 1), including ibuprofenoyl-CoA **1** and analogs **2** to **5** (13, 14), various straight and branched-chain fatty acyl-CoA esters (14, 15, 16, 17) **6** to **12**, and 2-arylthiopropanoyl-CoA esters (18). These acyl-CoA esters act as alternative substrates for AMACR (14, 19, 20) or prevent α-proton removal (14, 16) and hence formation of the enolate intermediate (1, 2, 5, 21). In addition, screening methods identified a number of pyrazoloquinolines and pyrazolopyrimidines (22), and non-specific protein derivatization agents (11) as inhibitors. Substrate-product inhibitors (23) and their aza analogs (24) have been identified as potent inhibitors of MCR. Although these have not been tested on human AMACR, they are expected to be good inhibitors.

**Figure 1:**
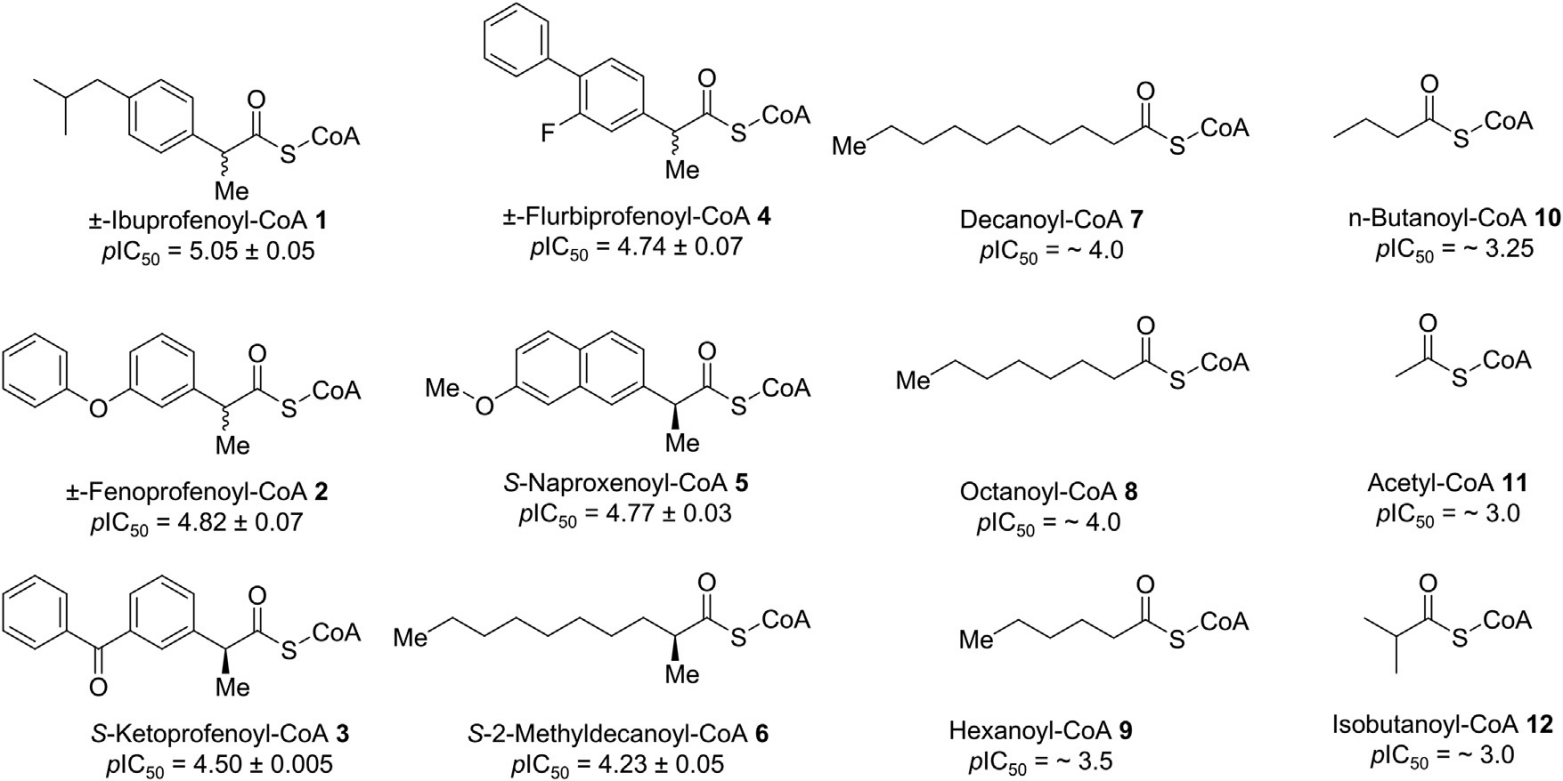
**Chemical structures and *p*IC_50_ values (mean ± SEM, n = 3) of acyl-CoA esters used in the present study.** Dose–response curves (26) were performed using the colorimetric (13) assay with 96 μM substrate, as previously described (13, 14, 18) (see Fig. 6*A* and inhibition assays in the Experimental procedures for further details).

No X-ray crystal structure of human AMACR 1A has been reported, and this has hampered optimization of inhibitors. In contrast, several X-ray crystal structures of the *M. tuberculosis* enzyme MCR in its apo form (6) or in complex with various acyl-CoA esters (5, 21) have been reported. These structures show that MCR belongs to the family III CoA transferases (5, 6). The catalytically active enzyme is a homodimer (with an apparent molecular weight of 83.6 or 89 kDa) with the active site at the interface between the two subunits with catalytic bases contributed by both subunits (5, 6, 7). The complexes with ibuprofenoyl-CoA **1** and 2-methyltetradecanoyl-CoA (2-methylmyristoyl-CoA) (5) reveal well-defined binding sites for the CoA ester and 2-methyl moieties. Catalysis was postulated to be mediated by deprotonation/reprotonation by the His126/Glu241 dyad or Asp156, with consequent movement of the substrate side-chain across the methionine-rich hydrophobic surface (5). A detailed understanding of how related compounds bind and the factors which determined binding strength are lacking as structures for only a single example of each class of substrates have been solved. However, it is known that substrate and inhibitor binding is related to (side-chain) lipophilicity for both AMACR (14, 18) and MCR (25). We recently reported the structure of wild-type MCR and key active site mutants from a new crystal form of the enzyme (7). In this report, we describe a second new crystal form for MCR and report the apo-enzyme structure. We also report structures of MCR in complex with ibuprofenoyl-CoA **1** and a series of analogs **2** to **5**, and a series of acyl-CoAs with alkyl side-chains **6** to **12** (Fig. 1). The structures reveal an alternative mechanism for the MCR-catalyzed reaction in which the *R*- and *S*- epimer side-chains are accommodated by subtle conformational changes (an “enantiomer superposition” model), rather than movement of the side-chain across the methionine-rich surface (“mirror image packing” model). We also describe the inhibitory activity of these acyl-CoAs (Fig. 1) using dose–response curves (26) to determine *p*IC_50_ values and rationalize their potencies based on structural information and physicochemical properties.

## Results and discussion

### Overall structure of wild-type MCR in a new form and with acyl-CoA complexes

**Figure 2:**
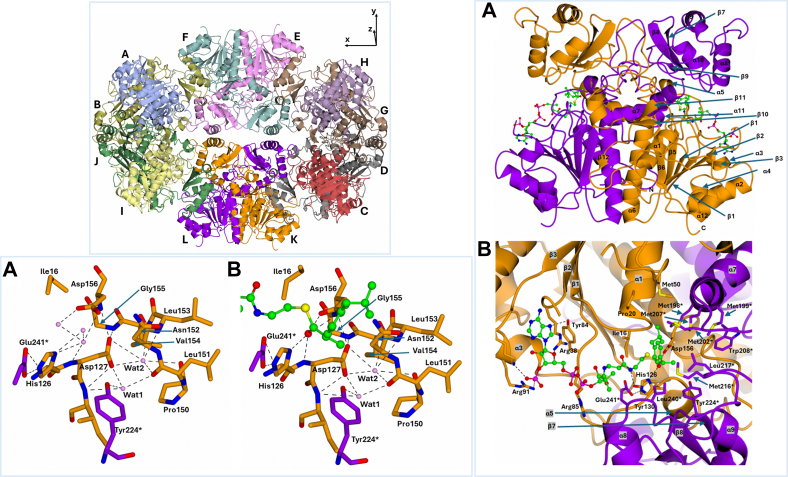
**Structural features of MCR.** (*Top left*) Crystal packing of 6 MCR homodimers in the I422 space group. The ribbon diagram shows 12 MCR molecules (*A–L*) in the asymmetric unit (AU) of the new crystal form (PDB code 9I2T). Each monomer is colored uniquely: i*ce blue, gold, pale crimson, gray, pink, sea green, pale brown, lilac, lemon, lawn green, dark orange, and purple*. (*Right*) Structure of the MCR homodimer with ibuprofenoyl-CoA **1** bound (PDB code 9I2U). *A*, overall homodimer structure, with subunit 1 in *dark orange* and subunit 2 in *purple*. The ligand is shown in *ball-and-stick* representation at the two active sites. Secondary structure elements were assigned using CCP4mg. *B*, close-up of the active site reveals an extended binding groove predominantly formed by residues from the large domain of one subunit; ligand-binding residues originate from several secondary structure elements. ∗Indicates a residue from the opposing subunit. (*Bottomleft*) Active site architecture and role of conserved water molecules. *A*, Apo MCR structure (PDB code 9I2T). *B*, ibuprofenoyl-CoA **1**–bound structure (PDB code 9I2U), with ligand in *green* (*ball-and-stick*). Subunit 1 is in *dark orange*, subunit 2 in *purple*, and conserved water molecules (Wat1 and Wat2) in *pink*. These waters participate in a hydrogen-bonding network essential for maintaining the integrity of the active site. ∗Indicates a residue from the other subunit.

Extensive crystallization screening of MCR (>1000 conditions) yielded a new crystal form suitable for ligand binding studies. This form obtained using 4 M ammonium acetate, 0.1 M Bis-Trispropane-ethanoic acid (pH 7.0), and crystallized in the tetragonal *I*422 space group. Wild-type MCR crystals diffracted to 2.40 Å resolution (data from Diamond Light Source, Didcot) and had unit cell dimensions a = 275.45 Å, b = 275.45 Å, c = 388.33 Å, with 6 homodimers (12 MCR subunits) in the asymmetric unit (Fig. 2, left top panel). All ligand-bound complex crystals belonged to the same space group, with nearly identical unit cell parameters and number of homodimers. Diffraction resolution for the ligand-bound structures ranged from 1.88 to 2.20 Å, selected based on CC_1/2_ values and the number of unique reflections at the highest resolution bin (27).

This crystal form differs from previously reported wild-type MCR structures: (a) form 1 (monoclinic C2, PDB: 1X74) and (b) form 2 (monoclinic C2, PDB: 8RMW), both of which had fewer molecules per asymmetric unit. Comparison of the 12 MCR molecules in the new asymmetric unit yielded RMS deviations of 0.43 to 0.49 Å, indicating high structural similarity (Fig. 2, right panel *A*). Compared to forms 1 and 2, the new structure also showed an RMS deviation of 0.48 to 0.55 Å over 357 C_α_ atoms.

Crystallographic statistics for the wild-type MCR and its 12 ligand-bound complexes are provided in Table 1. All 12 subunits within the asymmetric unit displayed high-quality electron density and preserved the characteristic MCR dimeric architecture. Each monomer comprises a large and a small domain (6, 7). The large domain (Met1–Ala188 and Arg331–Gly360) contains 7 helices and 7 β-strands forming a Rossmann-like fold (Fig. 2 right panel *A*). The small domain (Val189–Pro300) consists of 4 α-helices and 3 β-strands forming a distinct three-stranded antiparallel β-sheet. These domains are linked by an extended loop (Leu195–Tyr223), known as the “acyl binding region” due to its proximity to the acyl-CoA binding site (5).

**Table 1:** X-ray crystallographic data collection and refinement statistics (with RCSB-PDB codes)

	Wild-type MCR (9I2T)	Ibuprofenoyl-CoA 1 (9I2U)	Fenoprofenoyl-CoA 2 (9I2V)	*S*-ketoprofenoyl-CoA 3 (9I30)	Flurbiprofenoyl-CoA 4 (9I2W)	*S*-naproxenoyl-CoA 5 (9I2X)	S-2-methyl decanoyl-CoA 6 (9I2Z)
Beamline	I04	I04	I04	I04	I04	I04	I04
Wavelength used (Å)	0.9537	0.9537	0.9537	0.9537	0.9537	0.9537	0.9537
Crystallographic statistics							
Space group	I422	I422	I422	I422	I422	I422	I422
Unit-cell dimensions							
a, b, c (Å)	275.45 275.45 387.33	277.03 277.03 390.74	276.73 276.73 390.11	276.69 276.69 390.43	277.51 277.51 391.17	276.28 276.28 389.22	277.03 277.03 390.86
α, β, ϒ (°)	90.00 90.00 90.00	90.00 90.00 90.00	90.00 90.00 90.00	90.00 90.00 90.00	90.00 90.00 90.00	90.00 90.00 90.00	90.00 90.00 90.00
Resolution-range (Å)^a^	224.67 (2.40)	226.00 (2.17)	225.71 (2.07)	225.75 (2.08)	225.91 (2.20)	225.29 (2.00)	226.02 (2.11)
R_merge_^a^	0.40 (6.83)	0.28 (4.91)	0.19 (3.84)	0.30 (5.57)	0.41 (6.55)	0.25 (4.73)	0.27 (4.63)
R_pim_^a^	0.08 (1.30)	0.05 (0.97)	0.04 (0.74)	0.06 (1.14)	0.08 (1.31)	0.05 (0.92)	0.05 (0.94)
CC_1/2_^a^	1.00 (0.45)	1.00 (0.35)	1.00 (0.44)	1.00 (0.35)	0.99 (0.32)	1.00 (0.39)	1.00 (0.36)
Mean < I/σ(I) >^a^	7.60 (0.70)	10.40 (0.90)	14.00 (1.00)	7.90 (0.60)	7.10 (0.70)	10.10 (0.90)	10.00 (0.70)
Completeness (%)^a^	100.0 (100.0)	100.00 (100.00)	100.00 (100.00)	100.0 (100.00)	100.00 (100.00)	100.00 (100.00)	100.00 (100.00)
No. of observed reflections^a^	7,940,626 (401,528)	10,917,403 (508,843)	12,456,136 (624,071)	12,132,481 (543,028)	10,316,635 (478,360)	13,664,261 (665,006)	11,722,265 (533,063)
No. of unique reflections^a^	286,594 (14,101)	393,535 (19,347)	451,154 (22,181)	444,942 (21,839)	423,697 (20,833)	497,035 (24,424)	427,911 (21,060)
Multiplicity^a^	27.70 (28.50)	27.70 (26.30)	27.60 (28.10)	27.30 (24.90)	28.10 (29.00)	27.50 (27.20)	27.40 (25.30)
Refinement statistics							
R_work_/R_free_	0.21/0.25	0.20/0.24	0.21/0.25	0.22/0.25	0.21/0.24	0.21/0.24	0.20/0.24
RMS deviation in bond lengths (Å)	0.0089	0.0095	0.0097	0.0095	0.0097	0.0098	0.0098
RMS deviation in bond angles (°)	1.73	1.75	1.78	1.79	1.82	1.78	1.82
Ramachandran plot statistics (%)							
Favoured	91.39	93.62	93.98	93.90	93.74	94.82	94.60
Allowed	7.02	5.36	5.17	5.01	5.39	4.43	4.70
Outliers	1.59	1.02	0.85	1.09	0.87	0.75	0.70
Average B-Factors (Å^2^)							
Protein atoms	62.06	51.48	48.49	46.97	46.61	45.25	45.31
Ligand atoms	N/A	61.10	59.64	59.83	62.04	53.62	59.08
Water molecules	46.03	41.92	42.85	41.67	36.68	42.72	40.65
No. Atoms							
Protein	32,224	32,445	32,484	32,352	32,545	32,607	32,563
Ligand		744	780	792	780	768	720
Water	790	1264	1403	1505	1167	1793	1668


	Decanoyl-CoA 7 (9I36)	Octanoyl-CoA 8 (9I35)	Hexanoyl-CoA 9 (9I34)	Butanoyl-CoA 10 (9I33)	Acetyl-CoA 11 (9I31)	Isobutanoyl-CoA 12 (9I32)	
Beamline	I04	I04	I04	I04	I04	I04	
Wavelength used (Å)	0.9537	0.9537	0.9537	0.9537	0.9537	0.9537	
Crystallographic statistics							
Space group	I422	I422	I422	I422	I422	I422	
Unit-cell dimensions							
a, b, c (Å)	275.31 275.31 390.68	276.31 276.31 390.67	276.50 276.50 390.12	276.51 276.51 389.68	276.63 276.63 390.30	276.49 276.49 390.12	
α, β, ϒ (°)	90.00 90.00 90.00	90.00 90.00 90.00	90.00 90.00 90.00	90.00 90.00 90.00	90.00 90.00 90.00	90.00 90.00 90.00	
Resolution-range (Å)^a^	225.59 (2.08)	225.59 (2.08)	225.58 (1.95)	225.50 (2.02)	225.69 (1.88)	225.58 (1.94)	
R_merge_^a^	0.34 (4.11)	0.34 (4.11)	0.23 (3.98)	0.28 (5.70)	0.21 (4.86)	0.24 (4.23)	
R_pim_^a^	0.07 (0.83)	0.07 (0.83)	0.05 (0.71)	0.06 (1.09)	0.04 (0.93)	0.05 (0.82)	
CC_1/2_^a^	1.00 (0.389)	1.00 (0.39)	1.00 (0.41)	1.00 (0.30)	1.00 (0.31)	1.00 (0.33)	
Mean < I/σ(I) >^a^	9.20 (0.90)	9.20 (0.90)	12.60 (1.00)	11.10 (0.80)	11.50 (0.80)	12.50 (0.90)	
Completeness (%)^a^	100.00 (100.00)	100.00 (100.00)	100.00 (100.00)	100.00 (100.00)	100.00 (100.00)	100.00 (100.00)	
No. of observed reflections^a^	12,220,328 (549,303)	12,220,328 (549,303)	14,745,103 (729,035)	13,276,158 (668,578)	16,521,551 (828,009)	14,976,217 (744,676)	
No. of unique reflections^a^	443,965 (21,781)	443,965 (21,781)	538,030 (26,438)	483,899 (23,784)	600,791 (29,512)	546,383 (26,947)	
Multiplicity^a^	27.50 (25.20)	27.50 (25.20)	27.40 (27.60)	27.40 (28.10)	27.50 (28.10)	27.40 (27.60)	
Refinement statistics							
R_work_/R_free_	0.20/0.23	0.20/0.23	0.20/0.23	0.21/0.24	0.20/0.23	0.21/0.24	
RMS deviation in bond lengths (Å)	0.0099	0.0098	0.0104	0.0097	0.0104	0.0102	
RMS deviation in bond angles (°)	1.84	1.83	1.84	1.79	1.80	1.82	
Ramachandran plot statistics (%)							
Favoured	94.92	94.94	95.49	94.27	95.80	95.17	
Allowed	4.33	4.26	3.93	4.82	3.57	4.04	
Outliers	0.75	0.80	0.58	0.91	0.63	0.79	
Average B-Factors (Å^2^)							
Protein atoms	39.77	39.82	39.15	45.03	41.25	39.15	
Ligand atoms	48.61	46.60	45.44	53.18	48.00	44.66	
Water molecules	37.00	37.07	40.13	40.53	40.61	38.31	
No. Atoms							
Protein	32,487	32,572	32,593	32,654	32,608	32,681	
Ligand	708	684	660	636	1224	636	
Water	1692	1764	2235	1431	1894	2057	

a Values in parentheses are for the highest resolution shell.

Each MCR homodimer contains two active sites formed by residues from the large domain of one subunit and the small domain of the other (Fig. 2, right panel *A*). Key catalytic residues from the large domain include Asp156 (helix 6) and His126 (helix 5), while Glu241 (beginning of helix 9) is contributed by the small domain (Fig. 2, right panel *B*).

In all 12 acyl-CoA complex structures, clear ligand density was observed in the active site between His126 and Asp156, extending toward Arg38. In contrast, the wild-type structure showed only water molecules around these catalytic residues (Fig. 2 left lower panel). Ligands were modeled into the electron density using Fourier difference maps, with B-factors ranging from 47.1 to 66.3 Å^2^ (Table 2). The 2.4 Å wild-type structure includes 790 modelled water molecules; ligand-bound structures had more, consistent with their higher resolution (Table 1). Several water molecules appear to stabilize the active site upon ligand binding (Fig. 2 left lower panel).

**Table 2:** The B-factors for the 12 ligands bound to each MCR active site in the asymmetric unit

Monomer	1 (9I22U)	2 (9I2V)	3 (9I30)	4 (9I2W)	5 (9I2X)	6 (9I2Z)	7 (9I36)	8 (9I35)	9 (9I34)	10 (9I33)	11 (9I31)	12 (9I32)
A (Å^2^)	64.3	63.1	63.1	67.6	55.1	66.9	63.6	48.9	47.1	44.4	51.8	55.1
B (Å^2^)	61.1	58.8	56.3	62.5	50.6	56.2	63.3	52.4	47.4	51.8	46.7	49.4
C (Å^2^)	63.1	61.3	59.9	60.4	50.2	60.3	66.6	53.3	49.7	55.1	48.6	48.5
D (Å^2^)	56.5	55.9	53.0	58.8	45.0	58.7	61.9	46.2	41	46.9	45.8	46.0
E (Å^2^)	85.5	73.4	74.4	85.1	55.7	79.7	80	55.3	54.4	66.9	62.2	52.5
F (Å^2^)	58.5	60,0	55.7	60.9	50.2	63.3	67.1	64.1	51.6	54.5	51.5	52.1
G (Å^2^)	61.2	66.1	63.1	64.7	55.4	62.8	71.4	47.9	48.2	53.5	52.9	50.1
H (Å^2^)	86.2	76.2	72.7	89.4	58.4	76.4	85.2	56.6	49.8	69.7	60.5	54.6
I (Å^2^)	57.1	58.3	54.6	59.7	48.0	56.5	55.6	41.7	39.4	45.6	42.1	39.9
J (Å^2^)	53.9	56.6	50.6	56.9	46.9	56.7	62.2	55.5	45.8	49.3	41.6	45.2
K (Å^2^)	58.3	56.7	52.0	58	49.1	57.7	59.6	55.4	49.5	54.6	45.0	46.8
L (Å^2^)	61.0	56.8	57.1	60.7	47.1	59.2	58.6	43.2	41.4	42.6	42.7	39.9
Median (interquartile range) (Å^2^)	61.0 (57.4–64.0)	59.4 (56.7–65.4)	56.7 (53.4–63.1)	60.8 (59.0–66.9)	50.2 (47.3–55.3)	59.8 (56.9–66.0)	63.5 (60.2, 70.3)	52.9 (46.6–55.5)	47.8 (42.5–49.8)	52.09 (42.6–69.7)	47.5 (43.3–52.6)	48.1 (45.4–51.6)

Compound names and structures are given in Figure 1. The B-factors were calculated using the programme REFMAC5 (42).

### Structure of acyl-CoA substrate complexes

The structures of MCR complexes were determined by soaking active MCR crystals with a solution of ligand in the 0.30 to 0.91 mM range. All of them were highly similar to the unbound structure, with average RMS differences of 0.28 to 0.55 Å over 357 C_α_ atoms. Clear, unambiguous electron density for each ligand was observed in all 12 molecules within the asymmetric unit, and this density encompassed the CoA moiety, C_α_-Me group, thioester oxygen, and part or all of the side-chain. Each structure was refined with an occupancy of 1.0, with *R*- and *S*-epimers in a 1 to 1 ratio for chiral ligands. Ligand binding results in the movement of some key residues (Ile16, Arg38, Lys62, Arg85, Arg91) (Table 3).

**Table 3:** The distance of the ligand side-chain group to nearby/interacting residue side-chains

Residue	1 (9I2U)	2 (9I2V)	3 (9I30)	4 (9I2W)	5 (9I2X)	6 (9I2Z)	7 (9I36)	8 (9I35)	9 (9I34)	10 (9I33)	11 (9I31)	12 (9I32)
Ile16 (Å)	4.36	4.18	4.09	4.50	4.03	4.06	4.17	4.30	4.21	4.1	5.71	4.33
Met216∗ (Å)	4.59	4.25	3.55	4.42	4.96	4.48	3.51	3.83	4.08	6.93	7.32	5.27
Met198∗ (Å)	3.63	3.61	3.28	3.64	3.86	5.50	4.58	4.89	4.08	4.73	5.34	3.63
Met202∗ (Å)	4.86	5.27	5.22	5.16	5.09	6.11	4.64	5.16	6.76	7.93	7.49	6.72
Met207∗ (Å)	4.15	6.62	4.31	5.50	7.34	8.06	4.36	5.20	8.63	11.73	11.14	10.06
Gly17 (Å)	4.23	4.25	4.05	4.35	4.52	4.90	5.65	4.01	4.21	4.08	4.88	4.17
Leu217∗ (Å)	3.63	3.58	3.61	3.74	4.83	3.70	4.94	4.42	4.84	5.22	5.21	4.83
Leu240∗ (Å)	3.53	3.61	3.63	3.73	3.59	4.06	3.78	3.91	3.89	4.37	5.39	3.68
Met50 (Å)	6.85	7.35	6.75	7.15	7.47	9.35	8.64	9.40	7.79	8.92	8.88	7.40
Pro20 (Å)	7.65	7.50	7.26	7.70	8.39	8.68	7.95	8.22	8.09	8.62	9.02	7.92
Met199∗ (Å)	7.39	7.28	7.01	7.01	7.20	7.47	7.67	7.67	7.72	8.55	7.80	7.09
Trp208∗ (Å)	8.51	8.86	8.84	8.80	8.75	8.53	8.67	8.71	9.09	9.71	9.49	8.75

Compound names and structures are given in Figure 1. Distances in Å are based on the shortest distance from the side-chain residue of MCR to the side group of the ligand. ∗Represents a residue from the other subunit within a homodimer.

The MCR active site forms a long and deep extended binding groove (Fig. 2, left lower panel) which stretches in two opposite directions from the catalytic center (between His126 and Asp156) with the CoA moiety and side-chain binding on opposite sides. 2-Methylacyl-CoA esters bind the CoA moiety, C_α_-methyl group (C_α_-Me), thioester carbonyl oxygen, and side-chain in specific pockets within the active site. Ibuprofenoyl-CoA **1** and analogs showed little conformational heterogeneity, except at the terminal end of the side-chains. For the other ligands, the CoA moiety also showed little conformational heterogeneity, whilst the remainder of the molecule showed more variability, especially in the side-chain. In these structures (*e.g.*, hexanoyl-CoA **9**), the carbonyl oxygen atom formed a transient hydrogen bond with the amide nitrogen of Asp127 (2.53–4.26 Å). Two different conformations could be modelled for acetyl-CoA **11**, each at 0.5 occupancy.

### The CoA-binding pocket

The CoA binding pocket is formed entirely by residues from the large domain of one MCR subunit. The CoA moiety makes direct contact with nine residues and interacts indirectly with five others *via* bridging water molecules (Fig. 2, right panel *B*). These interactions are conserved across all complex structures and are consistent with earlier findings (5).

Key residues include Lys62, Arg85, and Arg91, which form salt bridges with the phosphate groups of the CoA ligand, while Ala59, Leu61, Tyr84, and Tyr130 participate in hydrogen bonding. Ile16 and Arg38 adopt alternative conformations compared to the native enzyme: Ile16 forms a hydrophobic contact with the ligand’s thioester sulfur, and Arg38 engages in π-stacking with the adenine ring. Arg85 also shifts to form a salt bridge with the pyrophosphate and hydrogen bonds with Asp295/B∗ and Gln122/A. Approximately 12 water molecules are displaced upon ligand binding, while others are conserved and mediate interactions with Met111, Gly83, Tyr130, Gly125, and Gln123 (Fig. 2, left lower panel).

### Thioester carbonyl oxygen binding pocket (the oxyanion hole)

This pocket lies adjacent to the CoA binding region and comprises catalytic residues His126 and Asp156, and the main-chain nitrogen of Asp127 (7). His126 forms a hydrogen bond with Glu241∗ (2.6–2.8 Å), and Asp156 interacts with Asn152 (2.9–3.3 Å), helping establish the active site's geometry (Fig. 2, left lower panel).

Clear electron density for the thioester carbonyl and C_α_-Me groups in the ibuprofenoyl-CoA **1** and *S*-2-methyldecanoyl-CoA **6** complexes shows a *cis* conformation. The thioester oxygen forms hydrogen bonds with Asp127, Asp156, and His126 (Fig. 2, left lower panel). Similar interactions are seen in other ligand complexes, although weaker due to ligand mobility. B-factors for the thioester carbonyl oxygen range from 38.4 to 71.0 Å^2^. These interactions define the “oxyanion hole,” proposed to stabilize the enolate intermediate and facilitate catalysis by lowering the p*K*a of the C_α_-H (5, 21, 25).

### The C_α_-methyl binding pocket

Positioned at the active site base between the thioester oxygen and side-chain binding pockets, this hydrophobic pocket comprises Leu217∗, Tyr224∗, and Ile240∗ from the second subunit. The C_α_-Me group also interacts with His126, Asp127, Asn152, and Asp156 (Fig. 2, right panel *B*). The geometry and interactions are consistent across all structures, with B-factors between 38.4 and 55.0 Å^2^. This pocket stabilizes both the side-chain conformation and the thioester carbonyl oxygen. The arrangement mirrors prior structures of ibuprofenoyl-CoA **1** and 2-methylmyristoyl-CoA (5).

### Geometry around the catalytic center

The catalytic center, formed from both MCR subunits, is structurally anchored by Asp127 and two conserved water molecules (Fig. 2, left lower panel). Asp127, part of helix 5, hydrogen bonds with His126 and Gly155, positioning His126 near Asp156. It also forms a hydrogen-bonding network with Wat1 and Wat2, which helps maintain active site geometry.

The region from Asn152 to Phe157 is preceded by a Pro149-Pro150-Leu151 sequence that forms a π-helix, a high-energy structural motif essential for active site organization (5, 28). This helix stabilizes catalytic and ligand-binding residues at the active site base.

### Side-chain binding pocket

Adjacent to the C_α_-Me pocket, this large hydrophobic cavity accommodates a variety of substrate side-chains. Most residues originate from the side-chain binding loop (Leu195–Tyr223) (23, 29), including Ile16, Gly17, Pro20, Met50, and multiple conserved methionines from the second subunit (*e.g.*, Met202∗, Met207∗, Met216∗). A conserved water molecule interacts with the amino group of Gly16 (Fig. 2, left lower panel *B*).

**Figure 3:**
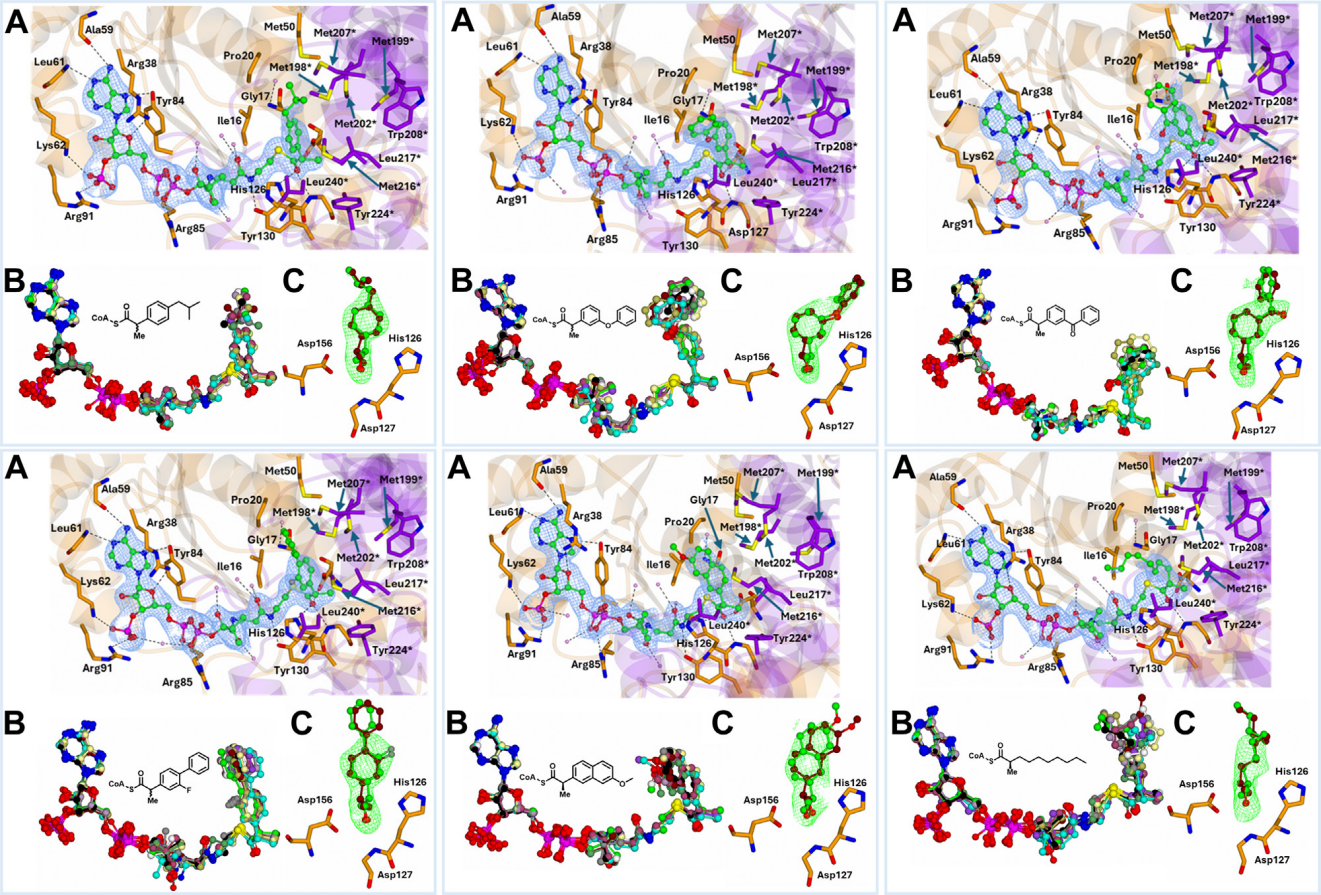
**Binding of acyl-CoA substrates into the MCR active site.** Structural and electron density analysis of 6 MCR-ligand complexes. *Each panel* shows the binding of a specific CoA thioester in the active site of MCR, with structural data from individual crystal structures as detailed below. For all panels: *A*, 2Fo-Fc electron density maps of the ligand-bound complexes contoured at 1σ. Residues from the large domain are colored *dark orange*, from the small domain *purple*. Water molecules are *pink*, and hydrogen bond and ionic interactions are indicated with dashes. ∗Indicates a residue from the other subunit of the homodimer. *B*, superposition of the 12 ligands within the asymmetric unit. *C*, unbiased Fo-Fc ligand density maps contoured at 3σ. Epimers (when present) are colored as indicated; the C_α_-H positions relative to Asp156 and His126 are highlighted. (*Top left*) Ibuprofenoyl-CoA **1** (PDB code: 9I2U); *R*- and *S-*epimers (*tan and green*). (*Top middle*) Fenoprofenoyl-CoA **2** (PDB code: 9I2V); *R*- and *S*-epimers (*tan and green*). (*Top right*) Ketoprofenoyl-CoA **3** (PDB code: 9I20); *R*- and *S*-epimers (*tan and green*). (*Bottom left*) Flurbiprofenoyl-CoA **4** (PDB code: 9I2W); *R*- and *S*-epimers (*green and cyan*). (*Bottom middle*) Naproxenoyl-CoA **5** (PDB code: 9I2X); *R*- and *S*-epimers (*tan and green*). (*Bottom right*) Methyldecanoyl-CoA **6** (PDB code: 9I2Z); *R*- and *S*-epimers (*tan and green*).

In 2-methylacyl-CoA structures, Fo–Fc maps did not favor *R*- or *S-*epimers; both were refined with full occupancy. The C_α_-H of each epimer points toward His126 or Asp156, resulting in distinct rotations around the C_α_ atom (Fig. 3).

In straight-chain acyl-CoA complexes, high B-factors in the thioester and CoA regions suggest ligand mobility. This displaces the thioester oxygen slightly from the oxyanion hole, resulting in transient hydrogen bonding to Asp127’s amide nitrogen (2.53–4.26 Å).

### Ibuprofenoyl-CoA 1 binding

The ibuprofenoyl-CoA **1** side group is a phenyl ring (C33, C34, C5, C6, C7, C8) with terminal carbons (C4, C3, C2, C1) at the C5 position. Here, the density completely covered the C_α_-ring and extended to some of the terminal carbons in most active sites (Fig. 3 top left). The terminal carbons showed variability in their orientation.

### Fenoprofenoyl-CoA 2 binding

Density for the fenoprofenoyl-CoA **2** side-chain covered the first aromatic ring (C25, C26, C27, C28, C29, and C36), the linking oxygen, and part of the terminal benzene ring (C35, C34, C33, C32, C31, and C30) (Fig. 3, top middle). The first and second benzene rings are not in the same plane, with the second ring rotated about the oxygen. Additionally, the terminal ring was oriented towards His126 in all active sites.

### Ketoprofenoyl-CoA 3 binding

The ketoprofenoyl-CoA **3** side-chain density extended to include the first aromatic ring (C25, C26, C27, C28, C29, and C37) and the ketone group (O18 and C30), while only part of the second ring (C31, C32, C33, C34, C35, and C36) had density. Like fenoprofenoyl-CoA 2, the terminal benzene ring is on the His126 side of the active (Fig. 3, top right). The terminal benzene ring shows conformational heterogeneity and is near the conserved methionine residues and forms a very close contact with these side chains.

### Flurbiprofenoyl-CoA 4 binding

The flurbiprofenoyl-CoA **4** ligand is unique in that it contains a single fluorine atom within its side-chain (Fig. 3 bottom left). Electron density for the side-chain group covered the first ring (C25, C26, C27, C28, C35, and C36) and the fluorine atom (F1). The second aromatic ring (C29, C30, C31, C32, C33, and C34) had minimal density due to high conformational heterogeneity. The fluorine was only on the His126 side of the active site and was close to Ile240^1^ (Fig. 3 bottom left).

### Naproxenoyl-CoA 5 binding

Naproxenoyl-CoA **5** has a unique structure with its naphthalene side-chain (Fig. 3, bottom middle). Density covers the first aromatic ring (C6, C7, C8, C33, C34, and C35) and most of the second ring (C2, C3, C4, C5, C34, and C35) while the terminal O-Me (O1, C1) had no electron density. The O-Me group was only on the His126 side of the active site.

### 2-Methyldecanoyl-CoA 6 binding

The 2-methyldecanoyl-CoA **6** side-chain group is a straight-chain group and electron density covered C8, C7, C6 but no density was observed (except in active site H) for the terminal carbons C5, C4, C3, C2, C1 due to conformational heterogeneity (Fig. 3, bottom right).

### Decanoyl-CoA 7 binding

**Figure 4:**
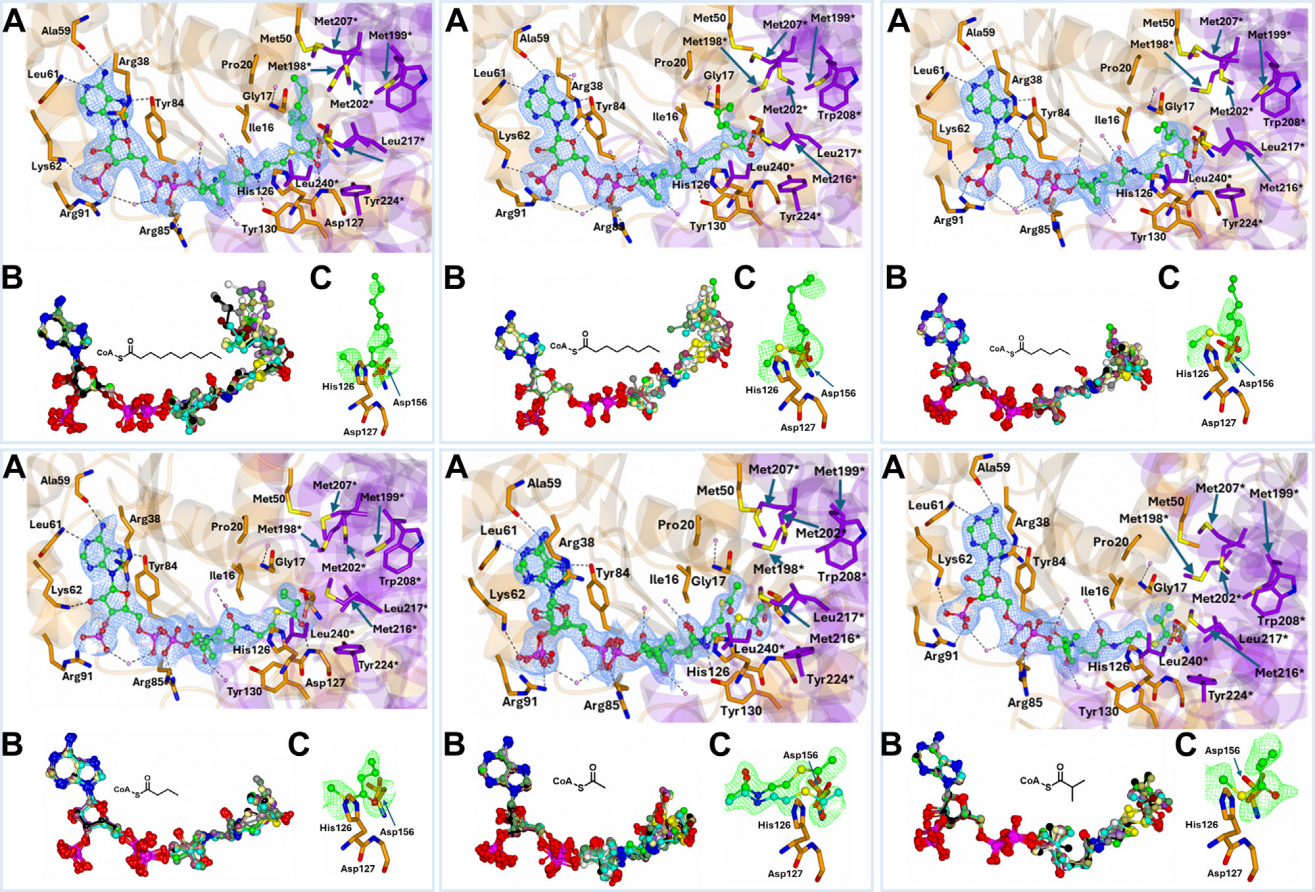
**Binding of acyl-CoA substrates into the MCR active site.** Structural and electron density analysis of 6 MCR-ligand complexes. *Each panel* shows the binding of a specific CoA thioester in the active site of MCR, with structural data from individual crystal structures as detailed below. For all panels: *A*, 2Fo-Fc electron density maps of the ligand-bound complexes contoured at 1σ. Residues from the large domain are coloured *dark orange*, from the small domain *purple*. Water molecules are pink, and hydrogen bond and ionic interactions are indicated with dashes. ∗Indicates a residue from the other subunit of the homodimer. *B*, superposition of the 6 ligands within the asymmetric unit. *C*, unbiased Fo-Fc ligand density maps contoured at 3σ. Epimers (when present) are colored as indicated; the C_α_-H positions relative to Asp156 and His126 are highlighted. (*Top left*) Decanoyl-CoA **7** (PDB code: 9I36). (*Top middle*) Octanoyl-CoA **8** (PDB code: 9I35). (*Top right*) Hexanoyl-CoA **9** (PDB code: 9I34). (*Bottom left*) n-Butanoyl-CoA **10** (PDB code: 9I33). (*Bottom middle*) Acetyl-CoA **11** (PDB code: 9I31). (*Bottom right*) Isobutanoyl-CoA **12** (PDB code: 9I32).

Decanoyl-CoA **7** is a straight-chain ester with a 10-carbon side chain. Electron density extends from C9 to C6 in most active sites, with few active sites such as E, F, and H having less coverage (C9 and C8 only) (Fig. 4, top left).

### Octanoyl-CoA 8 binding

Octanoyl-CoA **8** is a straight-chain ester with an 8-carbon straight-chain that constitutes the side-chain group. Electron density covers C7 and C6 while extending further in a few active sites, with active site B having the best electron density (Fig. 4, top middle).

### Hexanoyl-CoA 9 binding

Hexanoyl-CoA **9** is a straight-chain ester with a 6-carbon side chain. Electron density covered at least 3 of the carbons in the side-chain group, with active site D having be most coverage (Fig. 4 top right).

### n-Butanoyl-CoA 10 binding

n-Butanoyl-CoA **10** is a straight-chain CoA with a 4-carbon side-chain group. Electron density covers at least one of the side-chain carbons in all active sites and extends to all the carbons in some of the active sites (C, D, G, J, K, L) (Fig. 4 bottom left). There is heterogeneity in the conformation of the side-chain group.

### Acetyl-CoA 11 binding

Acetyl-CoA **11** ligand has the smallest side-chain group and has two conformations in all active sites (Fig. 4, bottom middle). One of these conformations (refined with 0.5 occupancy) has the side-chain group and the adjacent thioester oxygen close to Ile16. In this conformation, the carbonyl oxygen (O2) closest to the CoA sulfur (S1) atom is hydrogen bonded to 2 water molecules (Fig. 4 bottom middle). The other conformation (refined with 0.5 occupancy) is closer to the amide nitrogen of Asp127 and has its carbonyl oxygen (O2) hydrogen-bonded to Gly125 (2.53–3.05 Å) and Tyr130 (2.03–3.12 Å). Electron density for the CoA moiety was good, although density for the thioester oxygen and side-chain groups was not as easily interpreted due to the increase in flexibility in this region.

This acetyl-CoA **11** complex is similar to the previously reported structure (PDB: 2gd6) (5). Superimposing the 2 structures shows that the conformation of the CoA moieties is identical, while the conformation of the C_α_-Me and thioester oxygen groups is different. In the previously reported structure, a water molecule was identified in the density adjacent to Asp127, while the carbonyl oxygen was positioned above the water molecule, and the side-chain was adjacent to Ile16. This interpretation is different from that reported here, where the density adjacent to Asp127 is attributed to an alternative conformation.

### Isobutanoyl-CoA 12 binding

Isobutanoyl-CoA **12** is a short, branched-chain ester with a side-chain group made up of 3 carbons arranged such that there are 2 terminal CH_3_. Electron density covers part of this side-chain group in most active sites (Fig. 4, bottom right). This side-chain shows 3 distinct conformations although there is high flexibility. One of these conformations has the C_α_-H pointing toward the C_α_-Me binding pocket while the other conformations have the C_α_-H pointing towards Asp156 or His126, respectively. The thioester carbonyl oxygen and part of the CoA moiety group are difficult to interpret.

### The MCR catalytic mechanism

The MCR reaction consists of removal of the 2-methylacyl-CoA substrate α-proton (5, 7) to form an enolate intermediate (21). Isotope-labelling studies on human AMACR 1A [which has 41% primary sequence identity with MCR (6)] show that the reprotonation reaction is non-stereoselective to form a near 1 to 1 ratio of product epimers (19, 30, 31). His126 deprotonates the *S*-epimer, whilst Asp156 deprotonates the *R*-epimer, with the oxyanion hole stabilizing formation of the enolate intermediate (5). These new crystal structures are fully consistent with this mechanism.

**Figure 5:**
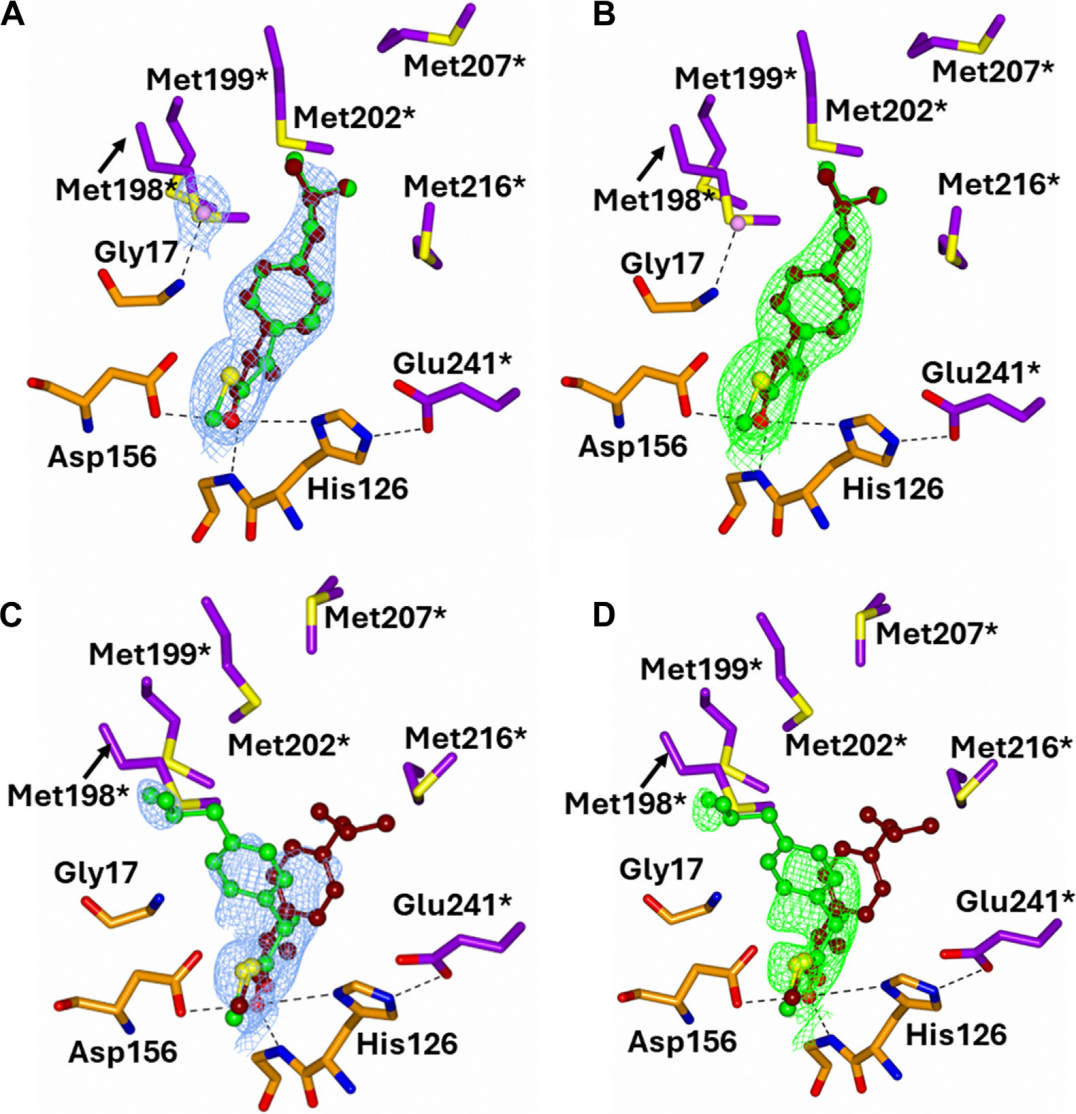
**The mechanism of MCR.** Panels (*A* and *B*) show the mechanism proposed in this study (PDB code 9I2U). Panels (*C* and *D*) show the mechanism proposed by Bhaumik *et al.* (5) (PDB code 2GCE). The *left-hand panels* (*A* and *C*) show the 2Fo-Fc electron density of ibuprofenoyl-CoA **1** contoured to 1σ. The *right-hand panels* (*B* and *D*) show the Fo-Fc electron density for the ligand contoured to 3σ. Residues from the large domain are in *dark orange* and those from the small domain are in *purple*, water molecules are in *pink*, and hydrogen bond and ionic interactions are shown as dashes. ∗Indicates a residue from the other subunit of the homodimer.

Previously, Bhaumik *et al.* proposed that the substrate and product side chains are accommodated in separate regions of the binding site (5), with the relative positions of α-proton and side-chain swapping places in the two epimers. Our results are consistent with a different mechanism in which the side-chain is held in a relatively static position with the different stereochemical configurations accommodated by a subtle clockwise or anticlockwise rotation of the α-carbon (Fig. 5). The crystal structures for ibuprofenoyl-CoA **1**, other 2-APA-CoA substrates **2-5**, and 2-methyldecanoyl-CoA **6** demonstrate that this mechanism applies to all these substrates.

A study by Sattar *et al.* reported that human AMACR 1A also catalyzes α-proton exchange of straight-chain acyl-CoA esters, albeit with reduced efficiency compared to branched-chain equivalents (32). Results presented herein show that MCR also catalyzes α-proton exchange of straight-chain acyl-CoA esters, although very low levels of exchange are observed. The reasons for this are apparent from the crystal structures of decanoyl-CoA **7** and the other straight-chain acyl-CoAs. The acyl ester moieties of these substrates are displaced relative to the oxyanion hole, with the α-protons not directly facing the catalytic bases. The side-chain conformations of these substrates are extremely heterogeneous, and this also likely accounts for the very low observed levels of exchange.

Unlike human AMACR (32), isobutyl-CoA **12** undergoes significant α-proton exchange. The MCR complex structure showed that three different conformations were observed. The most observed conformation (in active sites C, E, F, H, and K) had the α-proton pointing towards Asp156. A conformation in which the α-proton points towards His126 was also observed (in active sites G, I, and J) and towards the methyl binding pocket (in active sites A, B, and D). The latter conformation is consistent with the observation by Pal *et al.*, who noted that diphenylacetyl-CoA did not undergo α-proton exchange and postulated that this was due to binding of the α-proton in the methyl-binding pocket (23).

### MCR inhibition by acyl-CoA esters

**Figure 6:**
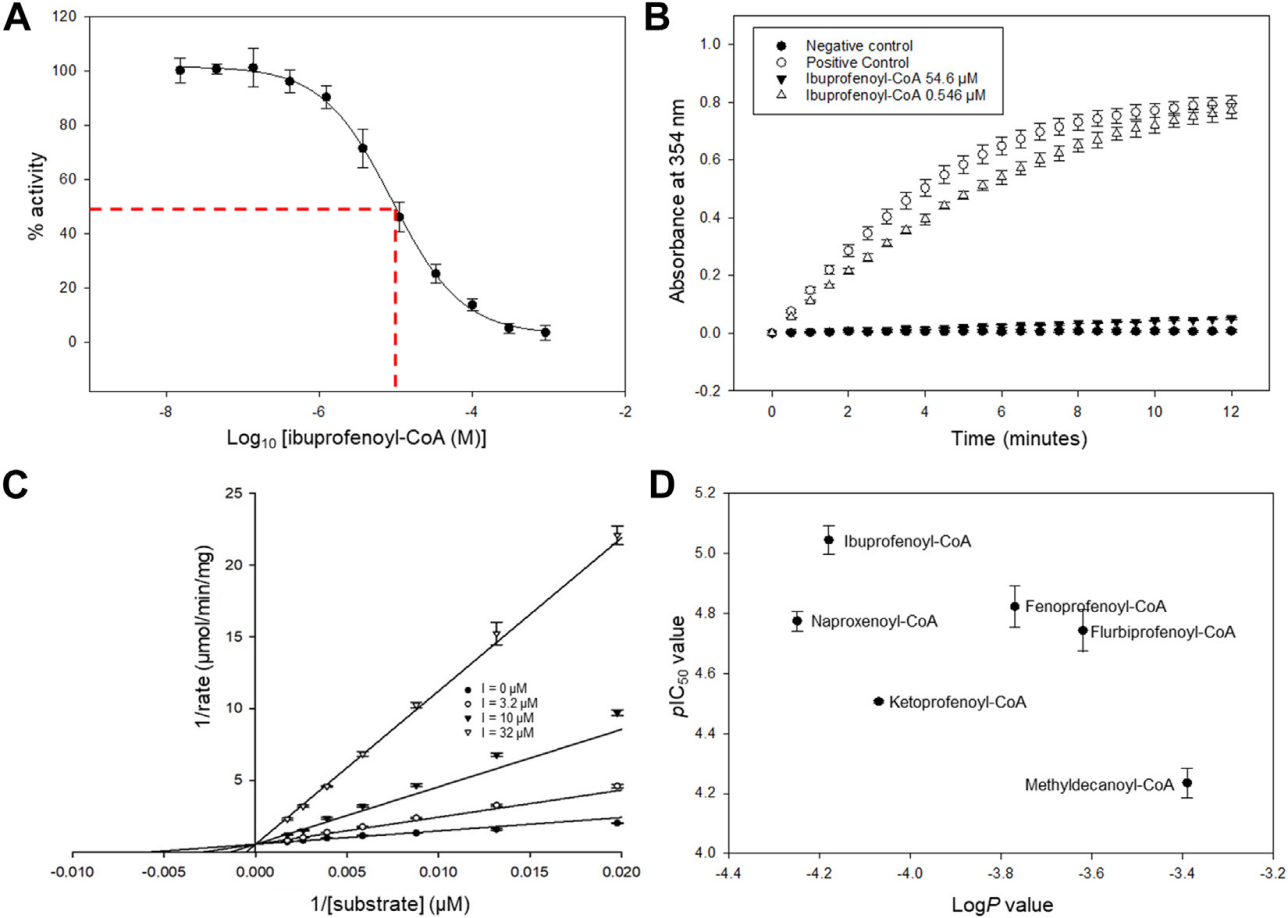
**Inhibition of MCR activity by acyl-CoA esters, as measured by the colorimetric assay**. *A*, a representative dose-response curve for ibuprofenoyl-CoA **1**. The *red dotted lines* show the log_10_ IC_50_ value for a representative independent repeat. Data are means ± SD (2 technical repeats). *B*, a jump dilution experiment showing reversible inhibition of MCR by ibuprofenoyl-CoA 1. Data are means ± SD for 3 technical repeats. *C*, a Lineweaver-Burk plot showing competitive inhibition of MCR activity by ibuprofenoyl-CoA **1**. Data are means ± SD (3 technical repeats) for a representative independent repeat. *D*, a plot of *p*IC_50_ value vs. calculated Log*P*. Data are means ± SEM (n = 3). See also Figs. S1, S13, and S18.

The colorimetric assay (7, 13) was used to measure inhibition of MCR activity by acyl-CoA substrates. Potency of inhibition was determined using does–response curves (Fig. 6*A*) to measure the *p*IC_50_ value. Ibuprofenoyl-CoA **1** and analogs and 2-methyldecanoyl-CoA **6** were relatively potent inhibitors, with ibuprofenoyl-CoA **1** displaying the highest levels of potency (*p*IC_50_ = 5.05 ± 0.05; mean ± SEM, n = 3). This level of potency is ∼20-fold less than the corresponding values for human AMACR, where ibuprofenoyl-CoA **1** and analogs had an IC_50_ value of 200 to 500 nM (13, 14) (*p*IC_50_ = 6.7–6.30, respectively). The determined potency for ibuprofenoyl-CoA **1** is about the same as for the product/substrate inhibitors reported by Pal *et al.* (23, 24). As expected, all these acyl-CoAs were reversible inhibitors (Fig. 6*B* and Figs. S13–S18) and caused competitive inhibition (Fig. 6*C* and Fig. S19, *A*–*G*) with a *K*_i_ value of 3.04 ± 0.34 μM (mean ± SEM, n = 3). This compares to 60.0 ± 4.78 nM for ibuprofenoyl-CoA **1** with human AMACR 1A (mean ± SEM, n = 1) (13). The *p*IC_50_ values for the straight-chain acyl-CoA esters and isobutanoyl-CoA **12** could not be accurately determined as sufficiently high inhibitor concentrations could not be achieved, but in all cases the *p*IC_50_ value was less than 4.0 (IC_50_ around 100 μM or higher). This means that the straight-chain acyl-CoA esters were about 10- to 100-fold less potent inhibitors than ibuprofenoyl-CoA **1**.

Previous experiments have demonstrated that inhibitor potency is positively correlated with calculated Log*P* values for AMACR (14, 18, 20) and MCR (23, 24, 25) inhibitors. We therefore performed a similar analysis for ibuprofenoyl-CoA **1** and analogs, and 2-methyldecanoyl-CoA **6** (Fig. 6*D*). This analysis showed that potency was negatively correlated with Log*P* for these compounds, that is potency was decreased with increasing Log*P* values. This is the opposite trend to that observed for human AMACR (14, 18) and the substrate-product MCR inhibitors (23, 24, 25). *S*-Ketoprofenoyl-CoA **3** and *S*-naproxenoyl-CoA **5** had *p*IC_50_ values approximately 0.3 lower than expected based on Log*P* (potency reduced by approximately two-fold). These two compounds have polar side-chain groups (the bridging carbonyl group and the methoxy group, respectively) protruding into the methionine-rich region of the hydrophobic binding site, which may cause a steric clash with nearby residues.

These observations provide a basis for the rational design of more potent inhibitors with side-chains that will bind strongly to the methionine-rich region whilst avoiding unfavorable polar interactions and steric clashes. Moreover, these results suggest that it may be possible to selectively inhibit MCR instead of AMACR by exploiting differences between the geometries of their side-chain binding pockets and by exploiting the hydrogen bond between the amide nitrogen of Gly17 and the conserved water molecule.

## Conclusion

Here we report the structures of the apo enzyme and in complex with 12 acyl-CoA esters along with testing of these acyl-CoAs as inhibitors, providing a framework for the development of novel inhibitors for the treatment of tuberculosis. There is a common binding mode for all acyl-CoAs. Ibuprofenoyl-CoA **1** and analogs bind relatively strongly to MCR (*p*IC_50_ = 4.50–5.05), although this is around 20-fold less potent than binding of the same compounds to human AMACR. In human AMACR, fenoprofenoyl-CoA **2** is the most potent inhibitor, with the other compounds having similar activities (14). In contrast, ibuprofenoyl-CoA **1** is the most potent inhibitor of MCR, and greater variation in inhibition is observed between the other compounds. Binding of acyl-CoA esters lacking an C_α_-Me group is much weaker than that observed in human AMACR 1A (13, 14, 32), and this appears to be related to the displacement of the ligand within the enzyme active site. There are a handful of key residues that contribute towards acyl-CoA binding. MCR can catalyze α-proton exchange for acyl-CoA substrates, although this is extremely inefficient [in contrast to human AMACR where straight-chain acyl-CoAs are relatively good substrates (32)].

Our structures confirm that there is a common catalytic mechanism, with deprotonation of acyl-CoAs by His126 and Asp-156. However, our results highlight that changes in 2-methylacyl-CoA epimeric configuration are accommodated by a rotation of the C_α_ with very little change in the side-chain conformation (Figure 3, Figure 4, Figure 5). This contrasts with the proposed mechanism of Bhaumik *et al.*, who proposed movement of the side chain across the methionine-rich surface (5) (Fig. 5). Reanalysis of the structures reported by Bhaumik *et al.*, shows electron density which can be interpreted as supporting our proposed mechanism. Given the high level of sequence identity between MCR and human AMACR 1A (6), it is likely that there is a common mechanism between these enzymes and those which are sequence related.

The new MCR crystal form enables facile complex formation, and this provides an ideal foundation for structure-based drug design. The 12 subunits within the asymmetric unit enable binding heterogeneity to be easily explored. The results presented in this paper demonstrate that MCR can bind acyl-CoA esters with a wide variety of side-chains. Further work will be required to understand the relationships between side-chain binding, measured potency, and physicochemical properties such as Log*P*. A significant drawback with using acyl-CoA esters is that they do not comply with Lipinski guidelines (33, 34) and hence they need to be delivered as pro-drugs (11, 15, 17, 18, 22, 35). Drug discovery approaches such as fragment-based, high-throughput, or virtual screening followed by optimization using structural approaches should enable development of small molecule drugs which are more compliant with Lipinski guidelines (33, 34) which do not require conversion to the acyl-CoA ester. Our new crystal form is ideal for structure-based development of hits discovered by these methods.

## Experimental procedures

General laboratory reagents were purchased from Merck (Gillingham) or Fisher Scientific and used without further purification. Molecular biology reagents were obtained from New England BioLabs, Stratagene, Promega or Novagen. The pET3a plasmid for wild-type MCR was synthesized by Genewiz (Takeley). Protein columns and resins were obtained from Cytiva Life Sciences. Crystallization screening conditions and equipment were sourced from Molecular Dimensions. The straight-chain acyl-CoA esters have been obtained from Larodan Lipids. Colorimetric substrate was synthesized as previously described (13). Ibuprofenoyl-CoA **1** and other 2-APA-CoAs **2** to **5** were synthesized as previously described (19). Solutions of substrate and inhibitors were quantified using ^1^H NMR and an internal standard. Briefly a stock solution of the sodium salt of 3-(trimethylsilyl)propionic-2,2,3,3-d_4_ acid (TMSP) in D_2_O was prepared. To quantify the colorimetric substrate and other acyl-CoAs 50 μl of the standard and 50 μl of the sample solution were combined and diluted with D_2_O (500 μl). ^1^H NMR spectra were recorded with a long relaxation delay (60s) to ensure reliable integration. The ratio of integrals for the CoA ester component of the inhibitors and colorimetric substrate (methyl signals near 1 ppm) were chosen for integration vs. the trimethylsilyl peak of TMSP and the ratio used to calculate the concentrations of the stock solutions. Stock solutions of acyl-CoA esters were vortex mixed for 5 min and centrifuged for 3 min at 13.3 k RPM before use in crystallization or kinetic experiments. Recombinant *M. tuberculosis* MCR was produced as previously described (36), buffer-exchanged into 10 mM potassium phosphate pH 8.8 and concentrated to 6 to 7.2 mg/ml before stored at −80 °C. All other reagents were procured through UK suppliers or agents.

### Crystallization and X-ray crystallographic study

Protein crystallization was carried out using the sitting drop and hanging vapor diffusion method at 22 °C. An Art Robbins Phoenix crystal screening nano-dispenser was used to screen 1056 different crystallization conditions from the following Molecular Dimensions crystallization screens: BSC, clear strategy screens, ligand-friendly screen, LMB-CS, JCSG+, Morpheus fusion screen, PACT, MIDAS +, PGA screen, SG1, structure screens 1 and 2. Each well of the 96-3 crystallization intelli-plates (Molecular Dimensions) contained 50 μl of reservoir solution, with recombinant wild-type MCR protein mixed with reservoir solution in a 2 to 1 or a 1 to 1 ratio to make up 0.3 μl drops. Plates were incubated for 5 weeks at 22 °C. These screens identified 8 conditions producing crystals which diffracted well, which were optimized using 24 well plates and the hanging drop method at 22 °C. Briefly, a 3 μl drop made up of a 2 to 1 ratio of protein to reservoir solution and a 2 μl drop with a 1 to 1 ratio was prepared and each incubated under 1 ml of reservoir solution. Crystals reached their maximum size after ∼4 weeks.

The crystals of wild-type MCR in complex with the acyl-CoA esters were obtained by soaking. Briefly, wild-type MCR crystals were transferred into 2 μl drops of reservoir solution containing 0.3 to 0.91 mM acyl-CoA and incubated under 500 μl reservoir for 24 h at 22 °C.-Soaked crystals were flash frozen using liquid nitrogen (100 K) and kept under a liquid nitrogen jet as diffraction images were collected on beamline I04 Diamond Light Source. 3600 X-ray diffraction images were collected at a 0.1° oscillation, 6.8 to 7.2 ms exposure time, 100.00% transmission and 8 to 12 MGy dose using the Eiger2 XE 16M detector (Dectris).

The highest resolution data sets were indexed and integrated using DIALS, then scaled using AIMLESS (CCP4i2 suite) (27, 37, 38). Solvent content was estimated using Matthew’s coefficient followed by structure solution by molecular replacement in PHASER with the aid of a previously reported MCR crystal structure (PDB code: 8RMW) (7). For the structures with the ligands, the acyl-CoA esters and their restraint libraries were built using ACEdrg (39) then manually added into the (Fo-Fc) Fourier difference electron density map using *COOT* (40, 41). *COOT* was also used for manual model building, while REFMAC5 was used for constrained refinement and the addition of water molecules (41, 42). The refined model was validated using Molprobity and PDB validation (37, 43, 44). Structural illustrations were prepared using CCP4mg (45).

### Deuterium-exchange assay

Exchange of substrate α-protons was assessed using the deuterium-exchange assay as previously described (19, 32). Assay mixtures contained 55 ng (2.34 nM) of active or heat-inactivated MCR, 100 μM acyl-CoA, 50 mM NaH_2_PO_4_-NaOH, pH 7.4 and *ca.* 85% (v/v) ^2^H_2_O in a total volume of 600 μl. Fenoprofenoyl-CoA 2 was used as a positive control. Reactions were initiated by the addition of enzyme, and reaction mixtures were incubated at 30 °C for 1 h before quenching at 55 °C for 10 min. NMR data were collected on a Bruker Avance Neo operating at 500.13 MHz for proton at 25 °C. Solvent suppression was achieved using a pre-saturation experiment. Typically, 128 transients were recorded. Data were processed using Topspin 4.0.7. Analysis of deuterium-exchange is based upon changes in intensity of either the substrate methine protons in the α-position or by the effects of the loss of those α-proton on a coupled methyl group.

### Inhibition assay

Inhibition assays were performed as previously described (13, 14, 18, 22) in 50 mM NaH_2_PO_4_-NaOH, 100 mM NaCl, and 1 mM EDTA, pH 7.4 and 3% (v/v) DMSO. Conversion of substrate to product was monitored at 354 nm. Final concentrations in the assay were MCR (3.12 nM), inhibitor (15.2 nM to 900 μM) and substrate (96 μM). Enzyme and inhibitor stock solutions were pre-incubated together for 10 min before initiating the assay. Rates were determined using ICEKAT (46, 47), with logarithmic fit of the data and correction of rates for delays between reaction initiation and first read. Dose-response curves plotted mean % activity vs. log_10_ M drug concentrations (26) using SigmaPlot 15.0 and data fitted to a 4-parameter logistic curve. Data are reported as *p*IC_50_ ± SEM (n = 3). Reversibility of inhibition was determined using a jump-dilution experiment (48) over a 12-min time-course using 31.2 nM enzyme, 96 μM substrate and inhibitor at 6⨯ or 0.06⨯ the IC_50_ value (Experiment 1 and 2, respectively).

### Kinetic assay

Data was collected as previously described using ibuprofenoyl-CoA **1** at 0, 3.2, 10, and 32 μM, 3.12 nM MCR, and 50.2 to 576 μM substrate (final concentrations) in 50 mM NaH_2_PO_4_-NaOH, 100 mM NaCl, and 1 mM EDTA, pH 7.4 and 3% (v/v) DMSO. Enzyme and inhibitor stock solutions were pre-incubated together for 10 min before initiating the assay. Rates were determined using ICEKAT (46, 47), with logarithmic fit of the data and correction of rates for delays between reaction initiation and first read. Data was analyzed using SigmaPlot 15.0 using Simple EK and the enzyme kinetics macro, with the correct inhibition model selected using graphs (Fig. S19, *A*–*G*) and statistical ranking of models. The determined *K*_i_ value is the mean ± SEM for three independent repeats.

## Data availability

The atomic coordinates and structure factors for all 13 structures [under accession codes - 9I2T (native), 9I2U (ibuprofenoyl-CoA **1** complex), 9I2V (fenoprofenoyl-CoA **2** complex), 9I30 (*S*-ketoprofenoyl-CoA **3** complex), 9I2W (flurbiprofenoyl-CoA **4** complex), 9I2X (*S*-naproxenoyl-CoA **5** complex), 9I2Z (*S*-2-methyldecanoyl-CoA **6** complex), 9I36 (decanoyl-CoA **7** complex), 9I35 (octanoyl-CoA **8** complex), 9I34 (hexanoyl-CoA **9** complex), 9I33 (n-butanoyl-CoA **10** complex), 9I31 (acetyl-CoA **11** complex), and 9I32 (isobutanoyl-CoA **12** complex) respectively] have been deposited in the RCSB Protein Data Bank, www.pdb.org.

## Supporting information

This article contains supporting information.

## Conflict of interest

The authors declare that they have no conflicts of interest with the contents of this article.

## Footnotes

∗ Indicates a residue from the other subunit of the homodimer. Standard three- and one-letter amino acid codes are used.

## References

[1] Lloyd M.D., Darley D.J., Wierzbicki A.S., Threadgill M.D. (2008). α-Methylacyl-CoA racemase: an “obscure” metabolic enzyme takes centre stage. FEBS J..

[2] Lloyd M.D., Yevglevskis M., Lee G.L., Wood P.J., Threadgill M.D., Woodman T.J. (2013). α-Methylacyl-CoA racemase (AMACR): metabolic enzyme, drug metabolizer and cancer marker P504S. Prog. Lipid Res..

[3] Lu R., Schmitz W., Sampson N.S. (2015). α-Methylacyl-CoA racemase provides *Mycobacterium tuberculosis* catabolic access to cholesterol esters. Biochemistry.

[4] Bhaumik P., Kursula P., Ratas V., Conzelmann E., Hiltunen J.K., Schmitz W. (2003). Crystallization and preliminary X-ray diffraction studies of an α-methylacyl-CoA racemase from *Mycobacterium tuberculosis*. Acta Crystallogr. D Biol. Crystallogr..

[5] Bhaumik P., Schmitz W., Hassinen A., Hiltunen J.K., Conzelmann E., Wierenga R.K. (2007). The catalysis of the 1,1-proton transfer by α-methyl-acyl-CoA racemase is coupled to a movement of the fatty acyl moiety over a hydrophobic, methionine-rich surface. J. Mol. Biol..

[6] Savolainen K., Bhaumik P., Schmitz W., Kotti T.J., Conzelmann E., Wierenga R.K. (2005). α-Methylacyl-CoA racemase from *Mycobacterium tuberculosis*: Mutational and structural characterization of the active site and the fold. J. Biol. Chem..

[7] Mojanaga O.O., Woodman T.J., Lloyd M.D., Acharya K.R. (2024). α-Methylacyl-CoA racemase from *Mycobacterium tuberculosis* - detailed kinetic and structural characterization of the active site. Biomolecules.

[8] Lee K.S., Park S.M., Rhee K.H., Bang W.G., Hwang K., Chi Y.M. (2006). Crystal structure of fatty acid-CoA racemase from *Mycobacterium tuberculosis* H37Rv. Prot. Struct. Funct. Bioinform..

[9] Zha S., Ferdinandusse S., Denis S., Wanders R.J., Ewing C.M., Luo J. (2003). α-Methylacyl-CoA racemase as an androgen-independent growth modifier in prostate cancer. Cancer Res..

[10] Kumar-Sinha C., Shah R.B., Laxman B., Tomlins S.A., Harwood J., Schmitz W. (2004). Elevated α-methylacyl-CoA racemase enzymatic activity in prostate cancer. Am. J. Pathol..

[11] Wilson B.A.P., Wang H., Nacev B.A., Mease R.C., Liu J.O., Pomper M.G. (2011). High-throughput screen identifies novel inhibitors of cancer biomarker α-methylacyl-coenzyme A racemase (AMACR/P504S). Mol. Cancer Ther..

[12] Takahara K., Azuma H., Sakamoto T., Kiyama S., Inamoto T., Ibuki N. (2009). Conversion of prostate cancer from hormone independency to dependency due to AMACR inhibition: involvement of increased AR expression and decreased IGF1 expression. Anticancer Res..

[13] Yevglevskis M., Lee G.L., Nathubhai A., Petrova Y.D., James T.D., Threadgill M.D. (2017). A novel colorimetric assay for α-methylacyl-CoA racemase 1A (AMACR; P504S) utilizing the elimination of 2,4-dinitrophenolate. Chem. Commun. (Camb.).

[14] Yevglevskis M., Lee G.L., Nathubhai A., Petrova Y.D., James T.D., Threadgill M.D. (2018). Structure-activity relationships of rationally designed AMACR 1A inhibitors. Bioorg. Chem..

[15] Carnell A.J., Hale I., Denis S., Wanders R.J.A., Isaacs W.B., Wilson B.A. (2007). Design, synthesis, and *in vitro* testing of α-methylacyl-CoA racemase inhibitors. J. Med. Chem..

[16] Carnell A.J., Kirk R., Smith M., McKenna S., Lian L.-Y., Gibson R. (2013). Inhibition of human α-methylacyl-CoA racemase (AMACR): a target for prostate cancer. ChemMedChem..

[17] Festuccia C., Gravina G.L., Mancini A., Muzi P., Di Cesare E., Kirk R. (2014). Trifluoroibuprofen inhibits alpha-methylacyl coenzyme A racemase (AMACR/P504S), reduces cancer cell proliferation and inhibits *in vivo* tumor growth in aggressive prostate cancer models. Anticancer Agents Med. Chem..

[18] Yevglevskis M., Nathubhai A., Wadda K., Lee G.L., Al-Rawi S., Jiao T. (2019). Novel 2-arylthiopropanoyl-CoA inhibitors of α-methylacyl-CoA racemase 1A (AMACR; P504S) as potential anti-prostate cancer agents. Bioorg. Chem..

[19] Woodman T.J., Wood P.J., Thompson A.S., Hutchings T.J., Steel G.R., Jiao P. (2011). Chiral inversion of 2-arylpropionyl-CoA esters by α-methylacyl-CoA racemase 1A (AMACR; P504S). Chem. Commun. (Camb.)..

[20] Yevglevskis M., Lee G.L., Threadgill M.D., Woodman T.J., Lloyd M.D. (2014). The perils of rational design – unexpected irreversible elimination of inorganic fluoride from 3-fluoro-2-methylacyl-CoA esters catalysed by α-methylacyl-CoA racemase (AMACR; P504S). Chem. Commun. (Camb.).

[21] Sharma S., Bhaumik P., Schmitz W., Venkatesan R., Hiltunen J.K., Conzelmann E. (2012). The enolization chemistry of a thioester-dependent racemase: the 1.4 Å crystal structure of a reaction intermediate complex characterized by detailed QM/MM calculations. J. Phys. Chem. B.

[22] Petrova Y.D., Wadda K., Nathubhai A., Yevglevskis M., Mitchell P.J., James T.D. (2019). Identification of novel small-molecule inhibitors of α-methylacyl-CoA racemase (AMACR; P504S) by high-throughput screening. Bioorg. Chem..

[23] Pal M., Khanal M., Marko R., Thirumalairajan S., Bearne S.L. (2016). Rational design and synthesis of substrate-product analogue inhibitors of α-methylacyl-coenzyme A racemase from *Mycobacterium tuberculosis*. Chem. Commun. (Camb.)..

[24] Pal M., Easton N.M., Yaphe H., Bearne S.L. (2018). Potent dialkyl substrate-product analogue inhibitors and inactivators of α-methylacyl-coenzyme A racemase from *Mycobacterium tuberculosis* by rational design. Bioorg. Chem..

[25] Lloyd M.D., Yevglevskis M., Nathubhai A., James T.D., Threadgill M.D., Woodman T.J. (2021). Racemases and epimerases operating through a 1,1-proton transfer mechanism: reactivity, mechanism and inhibition. Chem. Soc. Rev..

[26] Srinivasan B., Lloyd M.D. (2024). Dose-response curves and the determination of IC_50_ and EC_50_ values. J. Med. Chem..

[27] Evans P.R., Murshudov G.N. (2013). How good are my data and what is the resolution?. Acta Crystallogr. D Biol. Crystallogr..

[28] Kumar P., Bansal M. (2015). Dissecting π-helices: sequence, structure and function. FEBS J..

[29] Lu R., Schmitz W., Sampson N.S. (2015). α-Methyl acyl CoA racemase provides *Mycobacterium tuberculosis* catabolic access to cholesterol esters. Biochemistry.

[30] Darley D.J., Butler D.S., Prideaux S.J., Thornton T.W., Wilson A.D., Woodman T.J. (2009). Synthesis and use of isotope-labelled substrates for a mechanistic study on human α-methylacyl-CoA racemase 1A (AMACR; P504S). Org. Biomol. Chem..

[31] Woodman T.J., Lloyd M.D. (2023). Analysis of enzyme reactions using NMR techniques: a case study with α-methylacyl-CoA racemase (AMACR). Methods Enzymol..

[32] Sattar F.A., Darley D.J., Politano F., Woodman T.J., Threadgill M.D., Lloyd M.D. (2010). Unexpected stereoselective exchange of straight-chain fatty acyl-CoA α-protons by human α-methylacyl-CoA racemase 1A (P504S). Chem. Commun. (Camb.).

[33] Lipinski C.A., Lombardo F., Dominy B.W., Feeney P.J. (1997). Experimental and computational approaches to estimate solubility and permeability in drug discovery and development settings. Adv. Drug Deliv. Rev..

[34] Lipinski C.A., Lombardo F., Dominy B.W., Feeney P.J. (2001). Experimental and computational approaches to estimate solubility and permeability in drug discovery and development settings. Adv. Drug Deliv. Rev..

[35] Morgenroth A., Urusova E.A., Dinger C., Al-Momani E., Kull T., Glatting G. (2011). New molecular markers for prostate tumor imaging: a study on 2-methylene substituted fatty acids as new AMACR inhibitors. Chem. Eur. J..

[36] Mojanaga O.O., Acharya K.R., Lloyd M.D., Lloyd M.D. (2023). Modern Methods of Drug Design and Development.

[37] Agirre J., Atanasova M., Bagdonas H., Ballard C.B., Basle A., Beilsten-Edmands J. (2023). The CCP4 suite: integrative software for macromolecular crystallography. Acta Crystallogr. D Biol. Crystallogr..

[38] Waterman D.G., Winter G., Gildea R.J., Parkhurst J.M., Brewster A.S., Sauter N.K. (2016). Diffraction-geometry refinement in the DIALS framework. Acta Crystallogr. D Struct. Biol..

[39] Long F., Nicholls R.A., Emsley P., Grazulis S., Merkys A., Vaitkus A. (2017). AceDRG: a stereochemical description generator for ligands. Acta Crystallogr. D Biol. Crystallogr..

[40] Debreczeni J., Emsley P. (2012). Handling ligands with *Coot*. Acta Crystallogr. D Biol. Crystallogr..

[41] Emsley P., Lohkamp B., Scott W.G., Cowtan K. (2010). Features and development of *Coot*. Acta Crystallogr. D Biol. Crystallogr..

[42] Murshudov G.N., Skubák P., Lebedev A.A., Pannu N.S., Steiner R.A., Nicholls R.A. (2011). REFMAC5 for the refinement of macromolecular crystal structures. Acta Crystallogr. D Biol. Crystallogr..

[43] Chen V.B., Arendall W.B., Headd J.J., Keedy D.A., Immormino R.M., Kapral G.J. (2009). MolProbity: all-atom structure validation for macromolecular crystallography. Acta Crystallogr. D Biol. Crystallogr..

[44] Williams C.J., Headd J.J., Moriarty N.W., Prisant M.G., Videau L.L., Deis L.N. (2018). MolProbity: more and better reference data for improved all-atom structure validation. Protein Sci..

[45] McNicholas S., Potterton E., Wilson K.S., Noble M.E.M. (2011). Presenting your structures: the CCP4mg molecular-graphics software. Acta Crystallogr. D Biol. Crystallogr..

[46] Olp M.D., Kalous K.S., Smith B.C. (2020). ICEKAT: an interactive online tool for calculating initial rates from continuous enzyme kinetic traces. BMC Bioinf.

[47] Bursch K.L., Olp M.D., Smith B.C., Lloyd M. (2023). Modern Methods of Drug Design and Development.

[48] Copeland R.A., Basavapathruni A., Moyer M., Porter M. (2011). Impact of enzyme concentration and residence time on apparent activity recovery in jump dilution analysis. Anal. Biochem..

