# Supplementary material for: Molecular basis of acyl-CoA ester recognition by α-methylacyl-CoA racemase from Mycobacterium tuberculosis
Source: J Biol Chem. 2025 May 29;301(7):110302. doi: 10.1016/j.jbc.2025.110302 (PMC12246613; doi:10.1016/j.jbc.2025.110302)
Supplement: Supplementary Data [file mmc1.docx]

**SUPPLEMENTARY INFORMATION**

**Molecular basis of acyl-CoA ester recognition by α-methylacyl-CoA racemase from *Mycobacterium tuberculosis***

**Otsile O. Mojanaga, Timothy J. Woodman, Matthew D. Lloyd*, and K. Ravi Acharya***

*From the Department of Life Sciences, University of Bath, Claverton Down, Bath BA2 7AY, United Kingdom*

Footnote-

*For correspondence: K. Ravi Acharya, [bsskra@bath.ac.uk](mailto:bsskra@bath.ac.uk) or Matthew D. Lloyd, [M.D.Lloyd@bath.ac.uk](mailto:M.D.Lloyd@bath.ac.uk)

**Repeat 1 Coefficient Std. Error t P**

Min % 2.6168 2.5119 1.0418 0.3322

Max % 105.5482 1.6368 64.4859 <0.0001

LogIC_50_ -4.9613 0.0456 -108.7623 <0.0001

Hillslope -0.9604 0.0888 -10.8174 <0.0001

**Repeat 2 Coefficient Std. Error t P**

Min % 2.9187 1.8311 1.5940 0.1550

Max % 98.8641 1.2800 77.2363 <0.0001

LogIC_50_ -5.0486 0.0372 -135.6027 <0.0001

Hillslope -0.9180 0.0672 -13.6641 <0.0001

**Repeat 3 Coefficient Std. Error t P**

Min % 2.6510 2.2334 1.1870 0.2739

Max % 101.2444 1.7033 59.4411 <0.0001

LogIC_50_ -5.1260 0.0454 -112.8700 <0.0001

Hillslope -0.9734 0.0915 -10.6399 <0.0001

**Figure S1: Dose-response curve for ibuprofenoyl-CoA 1.** Data are mean % activity (2 technical repeats).

**Repeat 1 Coefficient Std. Error t P**

Min % -1.0173 5.6731 -0.1793 0.8628

Max % 99.3868 2.6087 38.0989 <0.0001

LogIC_50_ -4.6914 0.0959 -48.9214 <0.0001

Hillslope -0.8331 0.1364 -6.1061 0.0005

**Repeat 2 Coefficient Std. Error t P**

Min % 2.2137 2.6667 0.8302 0.4338

Max % 110.2399 1.5315 71.9798 <0.0001

LogIC_50_ -4.9315 0.0464 -106.1703 <0.0001

Hillslope -0.7292 0.0546 -13.3597 <0.0001

**Repeat 3 Coefficient Std. Error t P**

Min % -0.7388 5.8073 -0.1272 0.9023

Max % 93.4385 3.1627 29.5439 <0.0001

LogIC_50_ -4.8470 0.1114 -43.4946 <0.0001

Hillslope -0.8187 0.1589 -5.1533 0.0013

**Figure S2: Dose-response curve for fenoprofenoyl-CoA 2.** Data are mean % activity (2 technical repeats).

**Repeat 1 Coefficient Std. Error t P**

Min % -9.0253 4.2646 -2.1163 0.0721

Max % 100.4116 1.3543 74.1449 <0.0001

LogIC_50_ -4.5017 0.0670 -67.2371 <0.0001

Hillslope -0.6349 0.0506 -12.5470 <0.0001

**Repeat 2 Coefficient Std. Error t P**

Min % -2.7332 3.3753 -0.8097 0.4447

Max % 96.3493 1.1963 80.5372 <0.0001

LogIC_50_ -4.5116 0.0551 -81.8685 <0.0001

Hillslope -0.7776 0.0641 -12.1230 <0.0001

**Repeat 3 Coefficient Std. Error t P**

Min % -8.7722 6.7148 -1.3064 0.2327

Max % 103.0106 1.4595 70.5791 <0.0001

LogIC_50_ -4.2003 0.0928 -45.2463 <0.0001

Hillslope -0.7146 0.0761 -9.3931 <0.0001

**Figure S3: Dose-response curve for ketoprofenoyl-CoA 3.** Data are mean % activity (2 technical repeats).

**Repeat 1 Coefficient Std. Error t P**

Min % 1.7221 2.6256 0.6559 0.5328

Max % 97.7981 1.5915 61.4491 <0.0001

LogIC_50_ -4.8829 0.0493 -99.0301 <0.0001

Hillslope -0.9708 0.0972 -9.9861 <0.0001

**Repeat 2 Coefficient Std. Error t P**

Min % 0.8368 3.7864 0.2210 0.8314

Max % 95.9188 1.9109 50.1958 <0.0001

LogIC_50_ -4.6749 0.0651 -71.8352 <0.0001

Hillslope -1.0277 0.1397 -7.3540 0.0002

**Repeat 3 Coefficient Std. Error t P**

Min % 2.1053 6.4684 0.3255 0.7543

Max % 94.6575 3.2200 29.3969 <0.0001

LogIC_50_ -4.6728 0.1145 -40.8169 <0.0001

Hillslope -1.0055 0.2354 -4.2714 0.0037

**Figure S4: Dose-response curve for flurbiprofenoyl-CoA 4.** Data are mean % activity (2 technical repeats).

**Repeat 1 Coefficient Std. Error t P**

Min % -0.6400 4.5783 -0.1398 0.8928

Max % 99.5824 2.0877 47.7003 <0.0001

LogIC_50_ -4.7090 0.0787 -59.8197 <0.0001

Hillslope -0.7858 0.1003 -7.8338 0.0001

**Repeat 2 Coefficient Std. Error t P**

Min % -0.5309 0.9411 -0.5641 0.5903

Max % 102.2413 0.4875 209.7414 <0.0001

LogIC_50_ -4.8077 0.0163 -294.6531 <0.0001

Hillslope -0.8072 0.0225 -35.9351 <0.0001

**Repeat 3 Coefficient Std. Error t P**

Min % -2.7274 2.4764 -1.1013 0.3072

Max % 102.0390 1.2026 84.8512 <0.0001

LogIC_50_ -4.8091 0.0429 -112.1750 <0.0001

Hillslope -0.6833 0.0432 -15.8262 <0.0001

**Figure S5: Dose-response curve for naproxenoyl-CoA 5.** Data are mean % activity (2 technical repeats).

**Repeat 1 Coefficient Std. Error t P**

Min % 10.2466 5.4192 1.8908 0.1006

Max % 104.1285 2.1542 48.3381 <0.0001

LogIC_50_ -4.2021 0.0691 -60.8503 <0.0001

Hillslope -1.5837 0.3309 -4.7865 0.0020

**Repeat 2 Coefficient Std. Error t P**

Min % 13.4630 3.4688 3.8812 0.0060

Max % 101.8704 1.6941 60.1319 <0.0001

LogIC_50_ -4.1713 0.0437 -95.3974 <0.0001

Hillslope -2.4923 0.4537 -5.4932 0.0009

**Repeat 3 Coefficient Std. Error t P**

Min % 9.4607 3.5496 2.6653 0.0322

Max % 101.0137 1.5271 66.1479 <0.0001

LogIC_50_ -4.3325 0.0503 -86.1147 <0.0001

Hillslope -1.4587 0.2104 -6.9345 0.0002

**Figure S6: Dose-response curve for *S*-methyldecanoyl-CoA 6.** Data are mean % activity (2 technical repeats).


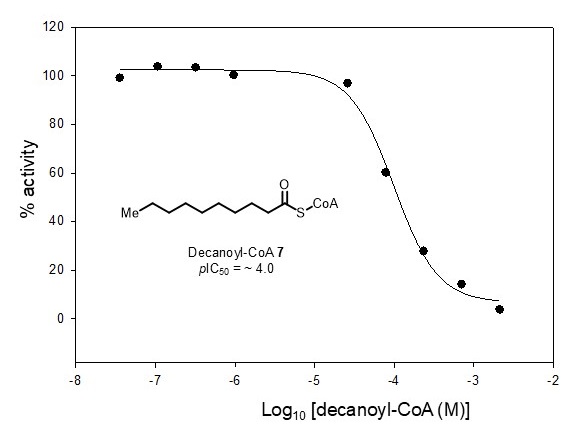


**Figure S7: Dose-response curve for decanoyl-CoA 7.** Data are mean % activity (2 technical repeats).


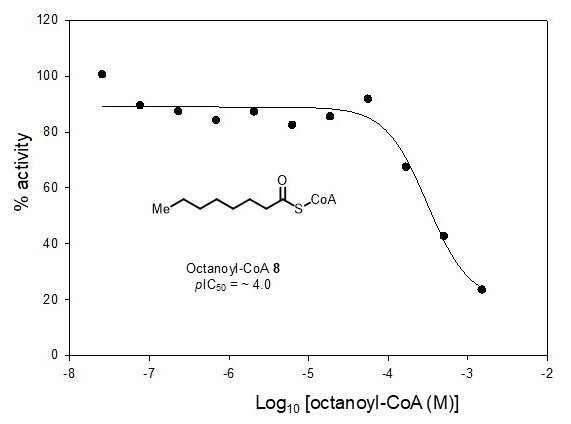


**Figure S8: Dose-response curve for octanoyl-CoA 8.** Data are mean % activity (2 technical repeats).

**Figure S9: Dose-response curve for hexanoyl-CoA 9.** Data are mean % activity (2 technical repeats).

**Figure S10: Dose-response curve for n-butanoyl-CoA 10.** Data are mean % activity (2 technical repeats).

**Figure S11: Dose-response curve for acetyl-CoA 11.** Data are mean % activity (2 technical repeats).

**Figure S12: Dose-response curve for isobutanoyl-CoA 12.** Data are mean % activity (2 technical repeats).

**Reversibility experiments**

**Figure S13: Jump-dilution experiment showing reversibility of inhibition by ibuprofenoyl-CoA 1.** Data are means ± SD (3 technical repeats).

**Figure S14: Jump-dilution experiment showing reversibility of inhibition by fenoprofenoyl-CoA 2.** Data are means ± SD (3 technical repeats).

**Figure S15: Jump-dilution experiment showing reversibility of inhibition by ketoprofenoyl-CoA 3.** Data are means ± SD (3 technical repeats).

**Figure S16: Jump-dilution experiment showing reversibility of inhibition by flurbiprofenoyl-CoA 4.** Data are means ± SD (3 technical repeats).

**Figure S17: Jump-dilution experiment showing reversibility of inhibition by ketoprofenoyl-CoA 5.** Data are means ± SD (3 technical repeats).

**Figure S18: Jump-dilution experiment showing reversibility of inhibition by ketoprofenoyl-CoA 6.** Data are means ± SD (3 technical repeats).

**Determination of *K*_i_ value for ibuprofenoyl-CoA 1**

±-Ibuprofenoyl-CoA **1**

**Figure S19A: A Direct Linear plot showing inhibition of enzymatic activity by ibuprofenoyl-CoA 1.** The coloured circles and inverted triangles show median estimates of *K*_m_ and *V*_max_.

**Figure S19B: A Direct Linear plot showing inhibition of enzymatic activity by ibuprofenoyl-CoA 1.** Data are means ± SD (3 technical repeats).

**Figure S19C: A Lineweaver-Burk plot showing inhibition of enzymatic activity by ibuprofenoyl-CoA 1.** Data are means ± SD (3 technical repeats).

**Figure S19D: A Eadie-Hofstee plot showing inhibition of enzymatic activity by ibuprofenoyl-CoA 1.** Data are means ± SD (3 technical repeats).


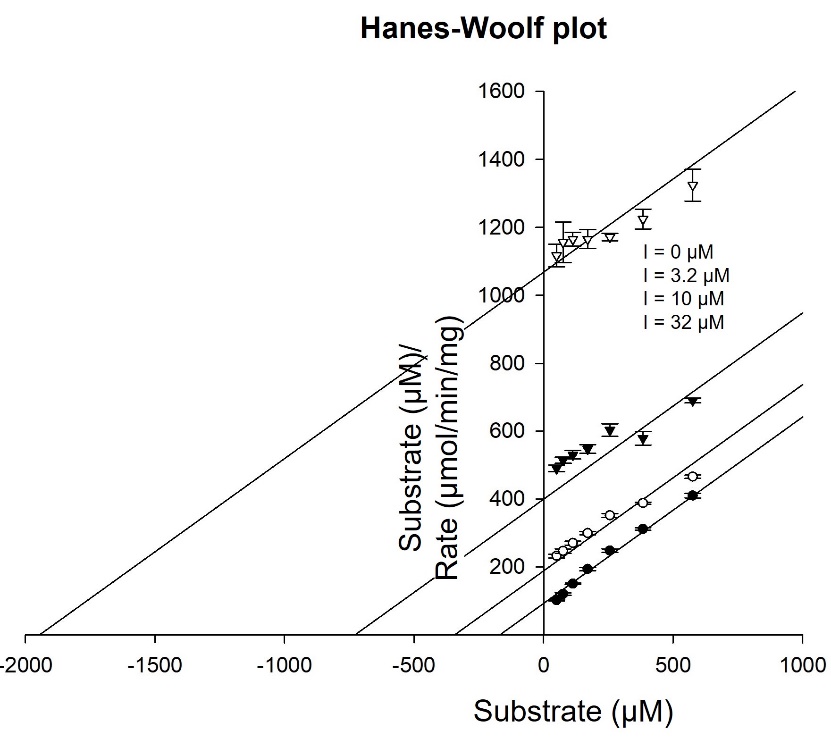


**Figure S19E: A Hanes-Woolf plot showing inhibition of enzymatic activity by ibuprofenoyl-CoA 1.** Data are means ± SD (3 technical repeats).

**Figure S19F: A residual plot of enzymatic activity as a function of substrate concentration.**

**Figure S19G: A residual plot of enzymatic activity as a function of inhibitor concentration.**

**Parameters (repeat 1)**

Value ±Std. Error 95% Conf. Interval

*V*_max_ (μmol/min/mg) 1.8229 0.04101 1.7413 to 1.9045

*K*_m_ (μM) 169.4368 9.6017 150.3319 to 188.5416

*K*_i_ (μM) 3.0584 0.1488 2.7624 to 3.3545

**Goodness of Fit**

Degrees of Freedom 81

AICc -524.531

R² 0.988

Sum of Squares 0.147

Sy.x 0.04.265

Runs Test p Value 0.500

**Data**

Number of x values 28

Number of replicates 3

Total number of values 84

Number of missing values 0

**Parameters (repeat 2)**

Value ±Std. Error 95% Conf. Interval

*V*_max_ (μmol/min/mg) 1.7201 3.843e-2 1.6436 to 1.7965

*K*_m_ (μM) 129.7242 7.7580 114.2879 to 145.1604

*K*_i_ (μM) 2.4440 0.1374 2.1707 to 2.7173

**Goodness of Fit**

Degrees of Freedom 81

AICc -524.531

R² 0.988

Sum of Squares 0.147

Sy.x 0.04.265

Runs Test p Value 0.500

**Data**

Number of x values 28

Number of replicates 3

Total number of values 84

Number of missing values 0

**Parameters (repeat 3)**

Value ±Std. Error 95% Conf. Interval

*V*_max_ (μmol/min/mg) 1.6852 6.535e-2 1.5551 to 1.8152

*K*_m_ (μM) 132.5213 13.8011 105.0609 to 159.9817

*K*_i_ (μM) 3.6156 0.3731 2.8732 to 4.3580

**Goodness of Fit**

Degrees of Freedom 81

AICc -524.531

R² 0.988

Sum of Squares 0.147

Sy.x 0.04.265

Runs Test p Value 0.500

**Data**

Number of x values 28

Number of replicates 3

Total number of values 84

Number of missing values 0
